# Supplementary material for: Regulation of Glucose Metabolism by MuRF1 and Treatment of Myopathy in Diabetic Mice with Small Molecules Targeting MuRF1
Source: Int J Mol Sci. 2021 Feb 23;22(4):2225. doi: 10.3390/ijms22042225 (PMC7926706; doi:10.3390/ijms22042225)
Supplement: Supplementary file 1 [file ijms-22-02225-s001.pdf]

Supplemental Figures

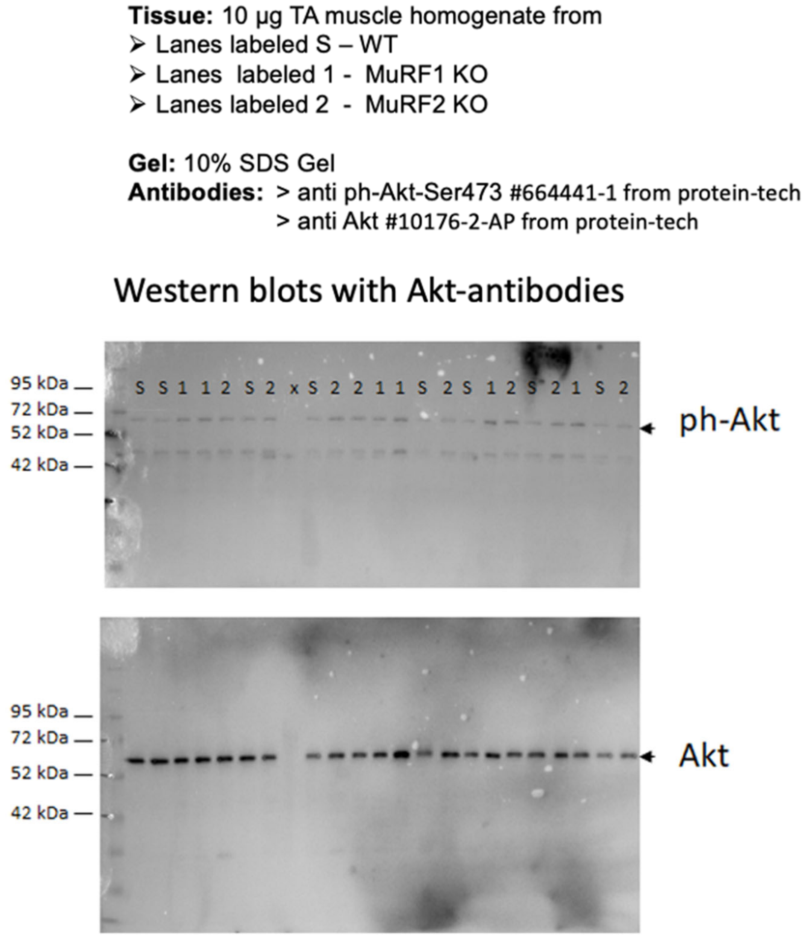

Supplemental Figure 1. ***Supporting information for Figure 3: Western blots with anti-Akt and anti-Foxo3a antibodies.***

Tissue: 10 µg TA muscle homogenate from
- Sham (S) WT
- > MuRF1 KO (1)
- MuRF2 KO (2)

Gel: 10% SDS Gel
Antibody: > anti ph-FOXO3A-Ser253 (ab#154786 from abcam)
> anti FOXA3A (PB9196 from Boster)

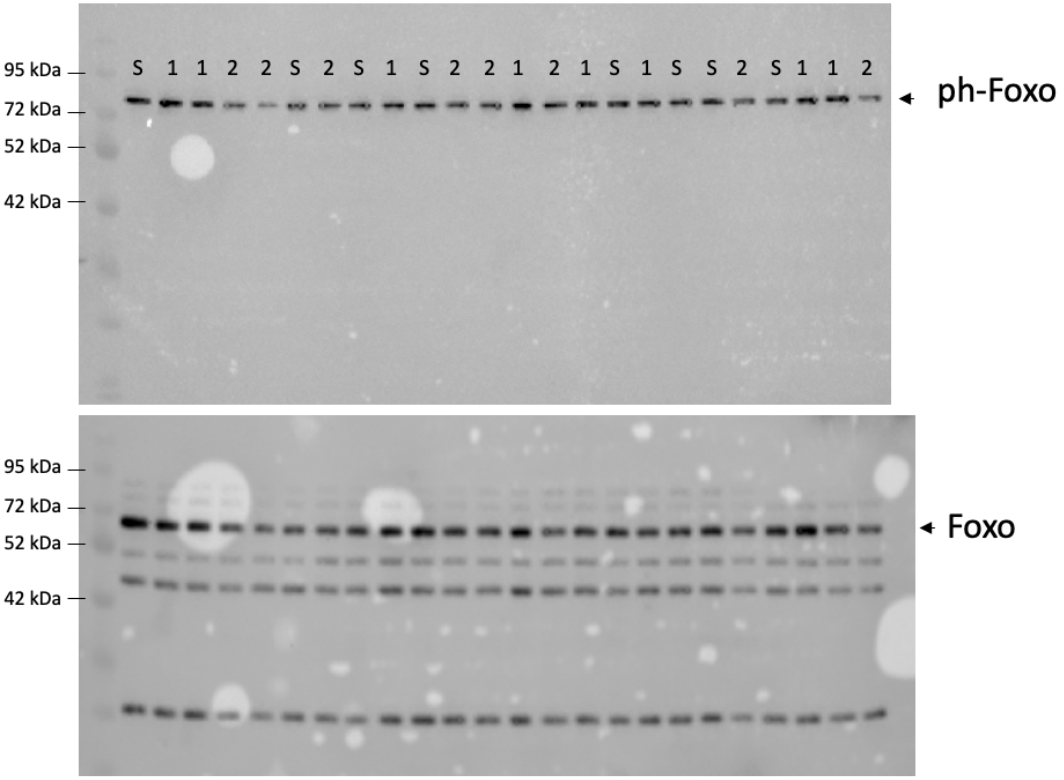

Supplemental Figure 1 continued. ***Supporting information for Figure 3. Western blots with anti-Akt and anti-Foxo3a antibodies.***

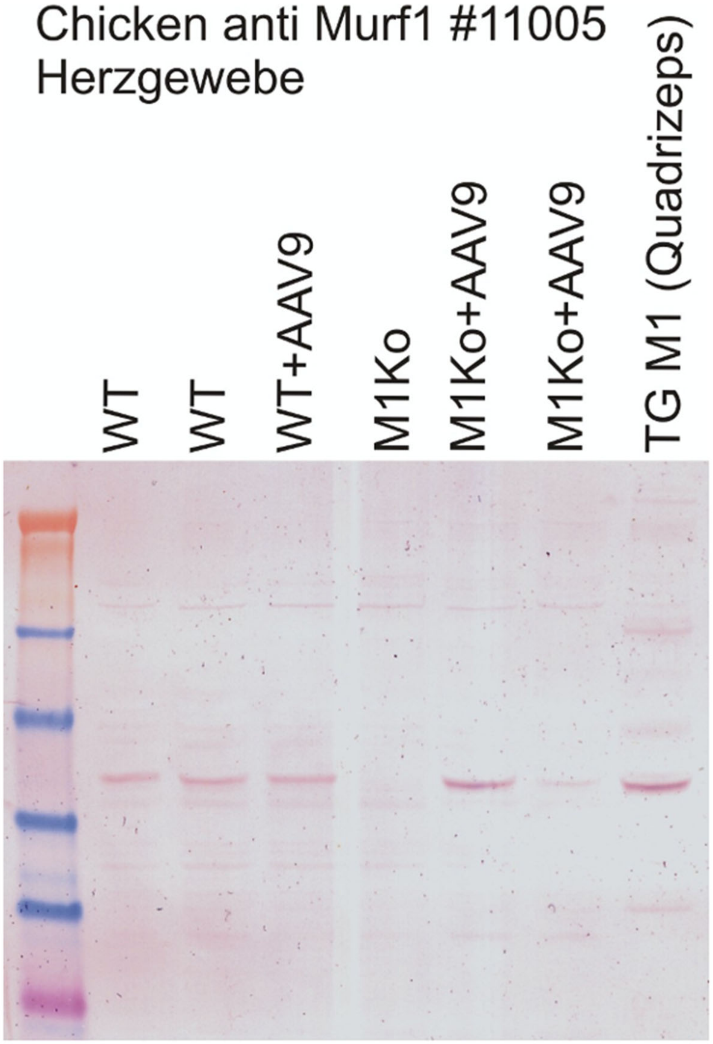

Supplemental Figure 2. ***Supporting information for Figure 4A. MuRF1-AAV9 particles were injected into tail veins, and myocardial extracts prepared from mouse hearts ("Herzgewebe"). MuRF1 was detected with a specific IgY-type antibody from chicken (lot#11005, home-made). Separation on a 6-10% gradient gel and blotting detected MuRF1 protein also in healthy WT hearts (left two lanes). Negative control: MuRF1-KO mice do not express MuRF1 in myocardium (MuRF1-KO lane). Injection of MuRF1-AAV9-MCL2 re-established myocardial MuRF1 expression (left lane MuRF1-KO + AAV9: after one month; next lane to the right, after 9 months). Positive control: QUAD from MuRF1-Tg mice. Lane most on the right "Quadrizeps". Size Marker: Rainbow marker from Amersham.***

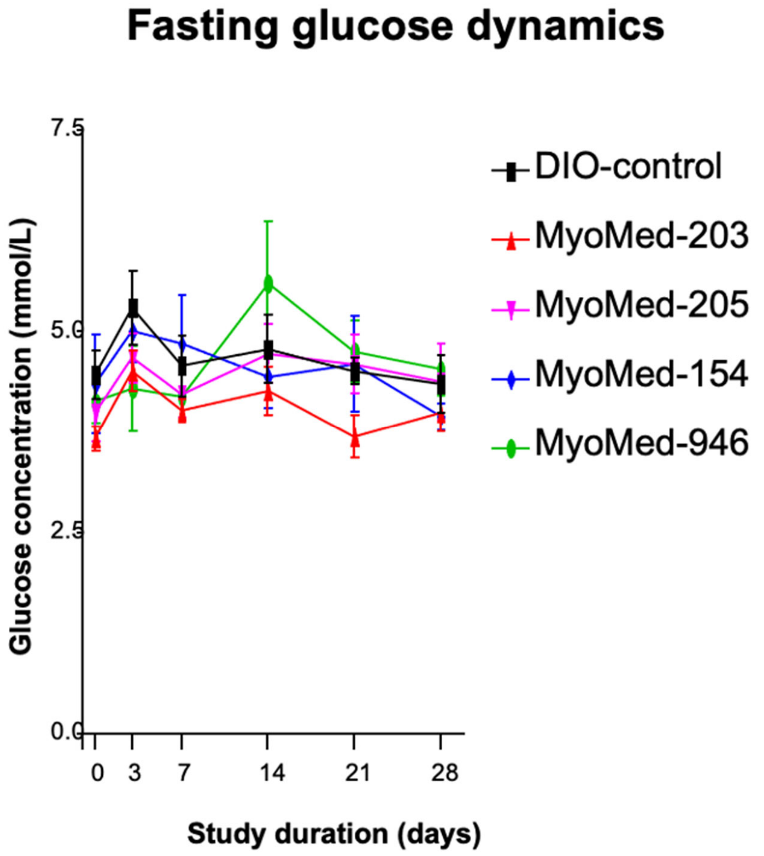

Supplemental Figure 3. ***Serum glucose in DIO mice during treatment with MuRF1-directed small molecules. During the 28 day-study protocol, serum glucose was monitored in DIO (green curve), and DIO-compound treated mice. There was no significant difference between the DIO group, Myomed-946 fed mice ("DIO-946", black curve), Myomed-205 fed mice ("DIO-205", yellow curve), Myomed-154- fed mice ("DIO-154", blue curve), or Myomed-203 fed mice ("DIO-203"), respectively.***

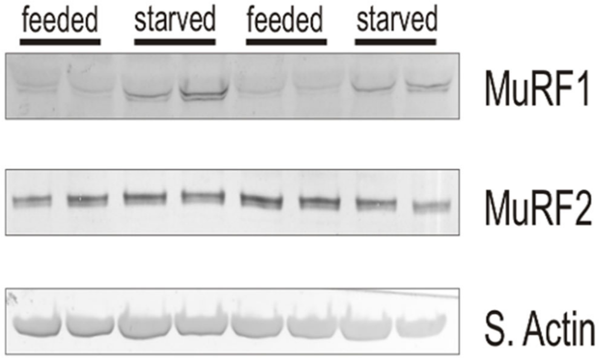

Supplemental Figure 4. ***Eight MuRF1 and eight MuRF2-KO mice were either normally fed or starved for 48 h with access to water. Western blots detect an Upregulation of MuRF1 protein in QUAD, whereas MuRF2 expression remains normal. Sarcomeric actin was used for normalization in this experiment.***
